# External validation of predictive models for prolonged postoperative ileus following colorectal resection

**DOI:** 10.1007/s00384-026-05152-4

**Published:** 2026-05-22

**Authors:** Camilo Ramírez-Giraldo, Bruno Cirillo, Martina Lucietto, Nicolò Fabbri, Antonio Biondi, Carlo Feo, Antonio Pesce

**Affiliations:** 1 Hospital Universitario Mayor-Méderi - Universidad del Rosario, Bogotà, Colombia; 2 Department of Surgery, Sapienza University of Rome, Viale Regina Elena 324, Rome, 00161 Italy; 3 Department of Surgery, University of Ferrara, Ferrara, Italy; 4 Department of General Surgery and Medical Surgical Specialties, University of Catania, Catania, Italy; 5 Department of Surgery, University of Ferrara - Azienda USL of Ferrara, Via Valle Oppio, 2, Lagosanto, FE 44023 Italy

**Keywords:** Colonic resection, Prolonged postoperative ileus, Predictive model, External validation, Perioperative period

## Abstract

**Introduction:**

Prolonged postoperative ileus (PPOI) is a frequent complication after colorectal surgery. Several prediction models have been proposed to estimate PPOI risk, but few have undergone external validation, limiting their generalizability and clinical usefulness. The primary aim of this study was to externally validate two predictive models for PPOI after colorectal resection; a secondary aim was to explore factors associated with PPOI in our cohort.

**Methods:**

Data from all consecutive patients who underwent elective colorectal resection in our department between 2019 and 2022 were retrospectively analyzed from a prospective database. Eligible criteria were age ≥ 18 years, elective colorectal resection, and ASA score I–III. Based on a recent systematic review, we selected the models by Hain et al. and Wolthuis et al. for external validation, as all required variables were available in our dataset. Model performance was assessed in terms of discrimination, calibration, and overall accuracy. An exploratory multivariable regression analysis was also performed to assess factors associated with PPOI.

**Results:**

Among 200 patients undergoing colorectal resection, 43 (21.5%) developed PPOI. Both prediction models showed poor external performance. The Hain model had a *C*-statistic of 0.597 (95% CI 0.514–0.681) and the Wolthuis model a *C*-statistic of 0.589 (95% CI 0.501–0.677), with suboptimal calibration and limited overall accuracy. In secondary exploratory multivariable analyses, postoperative opioid use was associated with PPOI in both models. In the model excluding postoperative oral intake initiation and autonomous postoperative mobilization, splenic flexure mobilization was associated with lower odds of PPOI; in the fully adjusted model, delayed postoperative oral intake initiation was associated with PPOI. These exploratory local findings should be interpreted as hypothesis-generating.

**Conclusions:**

External validation of two previously published PPOI prediction models demonstrated poor performance in this cohort, limiting their transportability to our setting. The secondary exploratory analysis identified potentially relevant postoperative factors, but these findings should be considered hypothesis-generating. Further research should prioritize harmonized PPOI definitions and robust multicentre validation of prediction models.

## Introduction

Prolonged postoperative ileus (PPOI) is a common condition after colorectal surgery, representing a delay in the return of normal bowel motility, and it typically persists beyond the 4th–5th postoperative day [1, 2]. It is distinct from physiological postoperative ileus, which resolves spontaneously within a few days. The onset of PPOI often leads to a longer postoperative hospital stay and may result in postoperative complications. Its incidence is still around 15–28% [3–5], highlighting how PPOI and the investigation of its determining and risk factors remain an ongoing clinical challenge. Several factors have been analyzed as potential risk factors for the occurrence of PPOI, including advanced age, male sex, preoperative anemia, surgical approach, opioid use, duration of surgery, manipulation of intestinal loops, and adherence to enhanced recovery protocols [4–6].

Multiple predictive models have been developed to estimate the risk of PPOI; however, only a few have undergone external validation, which limits their generalizability and clinical applicability [7]. An additional challenge is the heterogeneity in PPOI definitions across studies, as prediction models have often been developed using non-identical diagnostic criteria and outcome time windows. This lack of standardization may substantially affect model transportability, calibration, and discrimination when applied to external cohorts.

The primary objective of this study was to externally validate two predictive models for prolonged postoperative ileus following colorectal resection in our cohort. A secondary objective was to explore factors associated with PPOI occurrence in our population.

## Method

### Study design

Data from all consecutive patients who underwent colorectal resection in the surgical department of Azienda USL of Ferrara were retrospectively analyzed from a prospective database. A consecutive series of patients (*N* = 200) who underwent elective colorectal resection and completed a standardized Enhanced Recovery Protocol between 2019 and 2022 were included. Data were obtained from an anonymized database. Eligible criteria were as follows: age ≥ 18 years old, elective colorectal resections (right colectomy, transverse colectomy, left colectomy, sigmoidectomy, subtotal colectomy and anterior rectal resection), American Society of Anesthesiologists (ASA) score I–III. The exclusion criteria were as follows: TNM stage IV, inflammatory bowel disease, emergency surgery, patients with mid or low rectal cancer, including those who received neoadjuvant therapy, and patients undergoing abdominoperineal resection or other procedures involving perineal resection. These criteria were chosen to obtain a relatively homogeneous cohort of elective colorectal resections managed within a standardized ERAS pathway, although this restriction may have limited the inclusion of subgroups at higher baseline risk of PPOI. The study was conducted in accordance with the International Ethical Guidelines and the Declaration of Helsinki. All patients provided written informed consent before surgery, including consent for the use of anonymized clinical data according to institutional procedures. The study protocol (ID: 354/2019/Oss/AUSLFe) was approved by the local Ethics Committee (ComitatoEtico Area Vasta Emilia Centro (CE-AVEC)). This study is reported in accordance with the TRIPOD guidelines [8–10].

### Prediction models

A recent systematic review identified eleven risk prediction models to predict PPOI following colorectal surgery [5]. Based on this review, we examined the available models and variables and selected those described by Hain et al*.* [11] and Wolthuis et al*.* [12], because all required predictors were available in our dataset and the original model equations could be reconstructed without additional assumptions. This was therefore a pragmatic, feasibility-based selection rather than a systematic validation of the best-performing or most clinically relevant models identified in the literature.

The model proposed by Hain et al. [11] incorporates four variables: male sex, age ≥ 70 years, conversion to open surgery, and intra-abdominal surgical site infection, assigning one point to each if present. This model reported an area under the curve (AUC) of 0.715.

In contrast, the model developed by Wolthuis et al. [12]included five variables and it was based on the following formula: − 3.53 + 0.806 (if male) + 1.693 (if converted) + 1.399 (if open procedure) + 0.714 (if rectal resection) + 0.467 (if splenic flexure mobilization). This model reported an AUC of 0.72 (95% CI = 0.67–0.77). These two models included both perioperative and postoperative variables, which may limit their clinical usefulness for preoperative risk prediction.

### Outcomes variables

PPOI was the outcome variable predicted by the different models. In the model developed by Hain et al., PPOI was defined as the presence of abdominal distension and/or nausea and/or vomiting requiring nasogastric tube insertion during the postoperative period. In the model proposed by Wolthuis et al., PPOI was defined as the need for nasogastric tube insertion in patients presenting with nausea and at least two episodes of vomiting, who were unable to pass flatus or stool, accompanied by abdominal distension and absent bowel sounds.

In our study, PPOI was defined as the occurrence of two or more of Vather’s criteria [6] on or after the fourth postoperative day. In such cases, radiological confirmation was obtained using abdominal X-rays. Nasogastric tube (NGT) use was recorded throughout the entire postoperative hospital stay, including both intraoperative placement and any postoperative reinsertion. For outcome adjudication, only NGT reinsertion occurring on or after postoperative day 4 was considered consistent with PPOI criteria.

Morbidity was graded according to the Clavien-Dindo classification and included all complications that occurred until the hospital discharge or 30-day postoperatively [13].

### Statistical analysis

A descriptive analysis was performed using demographic, clinical, and surgical variables. Categorical variables were expressed as proportions, while continuous variables were summarized as medians with their interquartile ranges (IQR). Differences between groups, defined by the presence or absence of PPOI, were assessed using bivariate analyses. Categorical variables were compared using the chi-square test or Fisher’s exact test, as appropriate, and continuous variables were compared using the Mann–Whitney U test.

The discriminative ability of the logistic regression model was evaluated using the *C*-statistic and illustrated with a receiver operating characteristic (ROC) curve. Sensitivity and specificity were calculated at the optimal cutoff point, determined by Youden’s index. Overall predictive performance was additionally assessed using the Brier score. Model calibration was evaluated in the external validation cohort by comparing predicted probabilities from the original models with observed event rates. Calibration curves were constructed using a loess smoother applied to the predicted probabilities, and the 45° reference line was included to represent perfect calibration. Calibration intercept and slope were estimated using logistic recalibration models. Finally, the model’s goodness-of-fit was evaluated with the Hosmer–Lemeshow test.

Candidate predictors for the exploratory multivariable analysis were prespecified based on clinical relevance and prior literature. These included sex, splenic flexure mobilization, postoperative opioid use, time to postoperative oral intake initiation, and time to autonomous postoperative mobilization. Univariable screening using a threshold of *p* < 0.15 was applied solely to prioritize variables for inclusion, while clinically relevant variables were retained irrespective of statistical significance. Given the limited number of events, the multivariable models were kept parsimonious and considered exploratory.

Statistical significance was set at *p* < 0.05. All statistical analyses were conducted using R software (version 2025.05.0+496).

## Results

Between 2019 and 2022, a total of 200 consecutive colorectal resections were performed at our institution and included in the study. The median age of the patients was 74.0 (IQR = 66.0–81.2), and the majority were male (55.5%). Laparoscopic approach was used in 184 patients (92%) with a conversion rate of 6%. Of these, 43 patients (21.5%) developed prolonged postoperative ileus (PPOI). Demographic, clinical, and laboratory variables that differed significantly in the univariate analysis between patients with and without PPOI included the timing of postoperative oral intake initiation (*p* < 0.001) and the achievement of autonomous postoperative mobilization (*p* = 0.004). Regarding postoperative outcomes, patients with PPOI had longer hospital stays and longer intensive care unit stays. Additional patient characteristics by PPOI status are summarized in Table 1.

**Table 1:** Demographic, clinical, and surgical characteristics stratified by the presence or absence of PPOI

	*N* (%)	No PPOI (*n* = 157)	PPOI (*n* = 43)	*p* value
Age (median) (IQR) (years)	74.0 (66.0–81.2)	73.0 (66.0–81.0)	78.0 (65.5–83.0)	0.201
Sex				0.108
Female Male	89 (44.5) 111 (55.5)	75 (47.8) 82 (52.2)	14 (32.6) 29 (67.4)	
BMI (median) (IQR) (kg/m^2^)	26.1 (23.4–29.2)	26.3 (23.5–29.6)	24.9 (21.5–28.1)	0.180
ASA				0.900
I II III IV	1 (0.5) 71 (35.9) 125 (63.1) 1 (0.5)	1 (0.6) 55 (35.5) 98 (63.2) 1 (0.6)	0 (0.0) 16 (37.2) 27 (62.8) 0 (0.0)	
Comorbidity				
Diabetes mellitus Cardiovascular disease	35 (17.8) 134 (67.7)	28 (18.2) 102 (65.8)	7 (16.3) 32 (74.4)	0.950 0.377
Hemoglobin level (median) (IQR) (mg/dL)	12.1 (11.1–13.5)	12.1 (11.1–13.4)	12.4 (11.1–13.6)	0.975
Malnutrition status				0.220
Low risk Medium risk High risk	101 (69.2) 23 (15.8) 22 (15.1)	79 (72.5) 14 (12.8) 16 (14.7)	22 (59.5) 9 (24.3) 6 (16.2)	
Preoperative maltodextrin				0.658
Yes No	196 (98.0) 4 (2.0)	153 (97.5) 4 (2.5)	43 (100) 0 (0.0)	
Bowel preparation				0.526
No Yes	195 (97.5) 5 (2.5)	152 (96.8) 5 (3.2)	43 (0.0) 0 (0.0)	
Primary resection type				
Ileocecal resection Right hemicolectomy Transverse colectomy Left hemicolectomy Sigmoidectomy Subtotal colectomy Anterior rectal resection	2 (1.0) 107 (53.5) 8 (4.0) 25 (12.5) 40 (20.0) 13 (6.5) 5 (2.5)	2 (1.3) 83 (52.9) 4 (2.5) 23 (14.6) 33 (21.0) 9 (5.7) 3 (1.9)	0 (0.0) 24 (55.8) 4 (9.3) 2 (4.7) 7 (16.3) 4 (9.3) 2 (4.7)	1.000 0.864 0.118 0.135 0.636 0.623 0.292
Splenic flexure mobilization				0.115
No Yes	175 (87.5) 25 (12.5)	134 (85.4) 23 (14.6)	41 (95.3) 2 (4.7)	
Surgical approach				
Open Laparoscopic Conversion to open	4 (2.0) 184 (92.0) 12 (6.0)	4 (2.5) 145 (92.4) 8 (5.1)	0 (0.0) 39 (90.7) 4 (9.3)	0.658 0.970 0.505
Epidural analgesia				1.000
No Yes	128 (64.0) 72 (36.0)	100 (63.7) 57 (36.3)	28 (65.1) 15 (34.9)	
Intraoperative fluid administration (median) (IQR) (mL)	2500 (2000–3000)	2500 (2000–2900)	2500 (2250–3100)	**0.019**
Duration of surgery (median) (IQR) (min)	195.0 (165.0–225.0)	188.0 (165.0–221.0)	205.0 (175.0–236.5)	0.074
Postoperative oral intake initiation (median) (IQR) (days)	3.0 (2.0–4.0)	3.0 (2.0–3.0)	6.0 (5.0–8.4)	** < 0.001**
Postoperative opioid use				0.088
No Yes	105 (68.6) 48 (31.4)	83 (72.8) 31 (27.2)	22 (56.4) 17 (43.6)	
Autonomous postoperative mobilization (median) (IQR) (days)	3.0 (2.0–4.0)	3.0 (2.0–4.0)	4.0 (2.0–6.0)	**0.005**
Major postoperative complications (CD ≥ 3)				0.639
No Yes	191 (95.5) 9 (4.5)	151 (96.2) 6 (3.8)	40 (93.0) 3 (7.0)	
Postoperative hospital stays (median) (IQR) (days)	5.0 (4.0–7.0)	5.0 (4.0–7.0)	7.0 (6.0–14.0)	**< 0.001**
Postoperative intensive care unit stays (median) (IQR) (days)	0.0 (0.0–1.0)	0.0 (0.0–1.0)	1–0 (0.0–1.0)	**0.004**

Bold values indicate statistically significant *p* values (*p* < 0.05)

*BMI* body mass index, *ASA* american society of anesthesiologists, *IQR* interquartile ranges, *CD* Clavien-Dindo

**Fig. 1:**
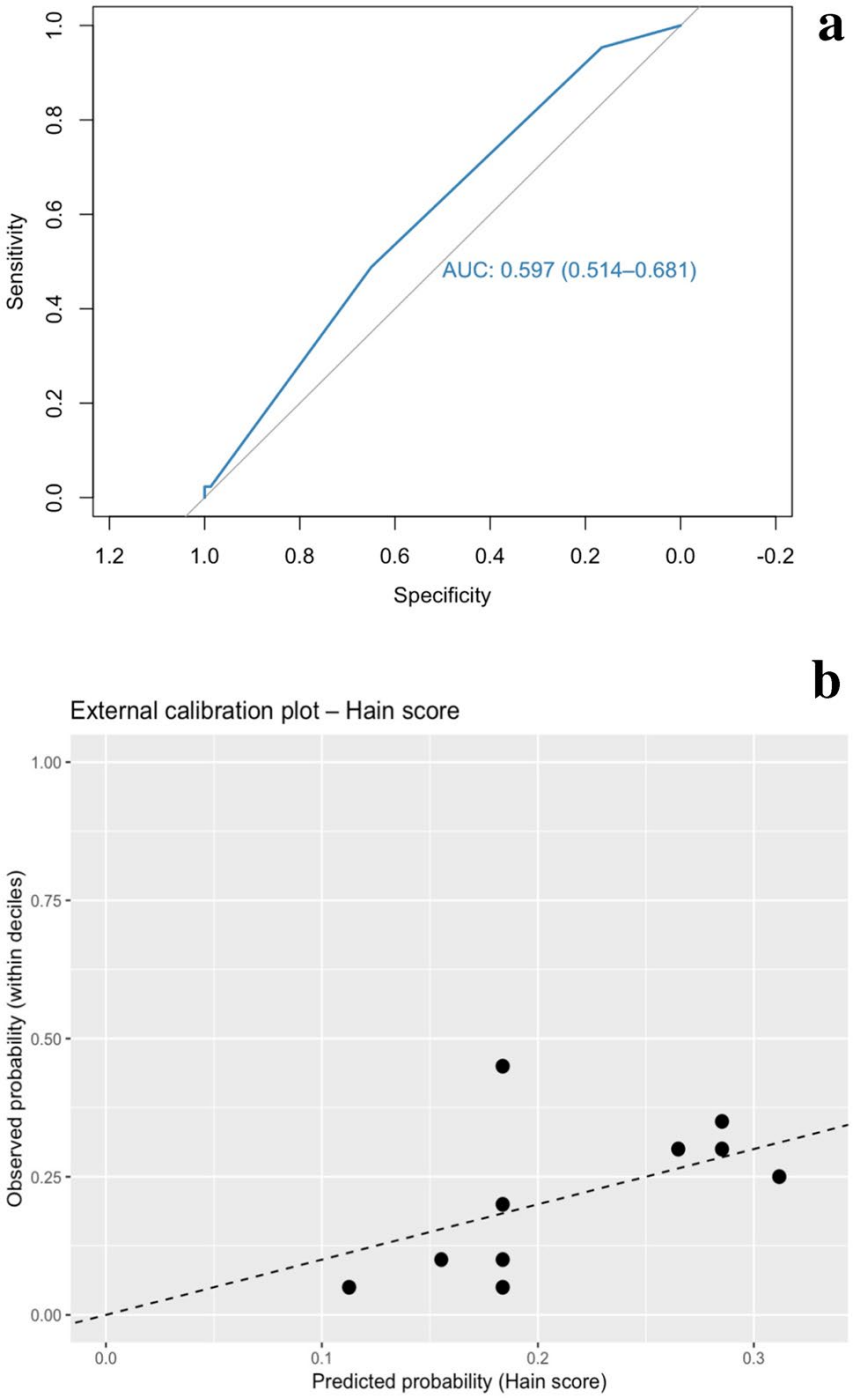
**A** ROC of the prediction model by Hain et al*.* **B** Calibration curves of the prediction model by Hain et al.

**Fig. 2:**
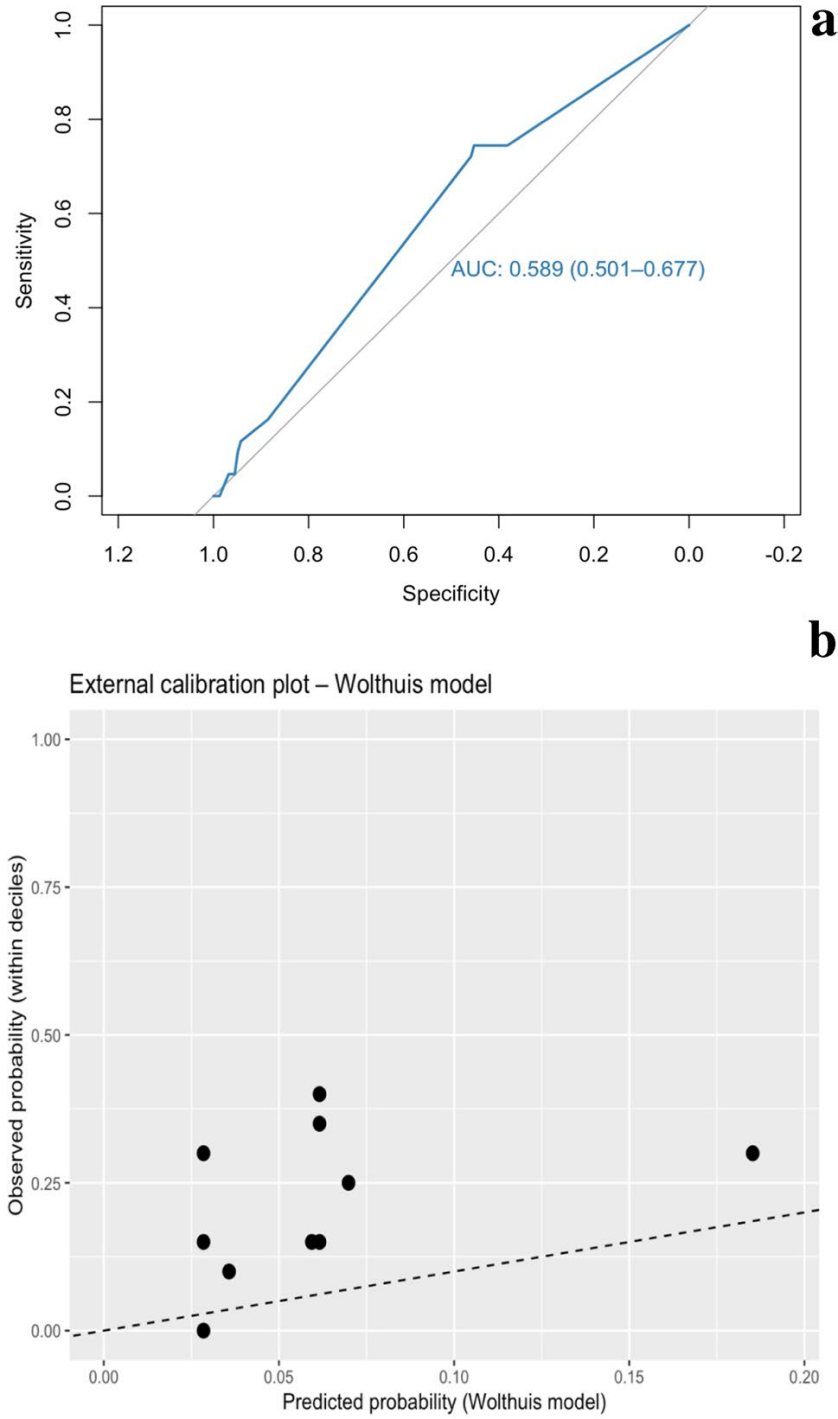
**A** ROC and calibration curves of the prediction model by Wolthuis et al*.* **B** Calibration curves of the prediction model by Wolthuis et al.

When evaluating the model proposed by Hain et al., we found its discriminatory ability to be poor (*C*-statistic = 0.597; 95% CI, 0.514–0.681) (Fig. 1A). The optimal cutoff point, determined by Youden’s index, was 1.5, corresponding to a sensitivity of 0.48 and a specificity of 0.64. The Brier score was 0.164, indicating limited overall accuracy. External calibration analysis demonstrated suboptimal performance. The calibration intercept was − 2.06, indicating that predicted risks were systematically higher than the observed event rate in our cohort (overall overestimation). The calibration slope was 0.57, suggesting that predictor effects were too extreme relative to the observed data. Figure 1B presents the bootstrap-corrected calibration curve, which visually confirms miscalibration across the predicted probability range. Although the Hosmer–Lemeshow goodness-of-fit test yielded a *p*-value of 0.303, this test is known to be sample-size dependent and was therefore interpreted cautiously. On the other hand, when evaluating the model proposed by Wolthuis et al*.*, we observed poor discrimination (*C*-statistic 0.589; 95% CI 0.501–0.677) (Fig. 2A). The optimal cutoff point, determined by Youden’s index, corresponded to a predicted probability threshold of approximately 5% (equivalent to a linear predictor value of − 2.9395), yielding a sensitivity of 0.74 and a specificity of 0.45. The Brier score was 0.167, also suggesting limited overall accuracy. In terms of calibration, the calibration intercept was − 0.11, indicating minimal systematic deviation in average predicted risk. However, the calibration slope was 0.41, demonstrating substantial attenuation of predictor effects and poor agreement between predicted and observed probabilities. Figure 2B illustrates the bootstrap-corrected calibration curve, confirming inadequate calibration across the risk spectrum. The Hosmer–Lemeshow goodness-of-fit test could not be computed using decile-based grouping due to limited distinct predicted probabilities. When evaluated using five groups, the test yielded a *p*-value of 0.146. However, given the known limitations and sample-size dependency of the Hosmer–Lemeshow test, this result was interpreted cautiously and in conjunction with calibration slope and graphical assessment.

Finally, as a secondary exploratory analysis, we performed a binary logistic regression analysis to explore factors associated with PPOI in our cohort. Two separate models were developed: one that included all variables of interest, including postoperative oral intake initiation and time to autonomous postoperative mobilization, and a second model that excluded both of these variables. This approach was taken because, although early oral intake and early mobilization are recommended in current enhanced recovery after surgery (ERAS) protocols to reduce complications such as PPOI, delays in either may also represent consequences or early manifestations of PPOI rather than true antecedent factors. In the model including all variables, both a longer time to postoperative oral intake initiation and postoperative opioid use were associated with PPOI. In the model excluding postoperative oral intake initiation and time to autonomous postoperative mobilization, postoperative opioid use was associated with PPOI, whereas splenic flexure mobilization was associated with lower odds of PPOI, while male sex and goal-directed fluid therapy showed non-significant trends (Table 2*)*. These findings should be interpreted as exploratory and hypothesis-generating.

**Table 2:** Binary logistic regression model to identify factors related to PPOI

	Model 1		Model 2	
	OR (95% CI)	*p* value	OR (95% CI)	*p* value
Sex		0.090		0.882
Female Male	Reference 1.96 (0.91–4.37)		Reference 1.11 (0.28–4.44)	
Splenic flexure mobilization		**0.045**		0.125
No Yes	Reference **0.20 (0.03–0.79)**		Reference 0.15 (0.01–1.24)	
Autonomous postoperative mobilization (days)	-	-	0.79 (0.53–1.09)	0.232
No opioid use		**0.027**		**0.002**
No Yes	Reference **0.40 (0.17–0.90)**		Reference **0.10 (0.02–0.41)**	
Intraoperative fluid administration (mL)	1.00 (1.00–1.00)	0.079	1.00 (1.00–1.00)	0.651
Postoperative oral intake initiation (days)	-	-	**6.54 (3.41–16.10)**	< 0.001

Bold values indicate statistically significant *p* values (*p* < 0.05)

*OR* odds ratio

## Discussion

PPOI is one of the main causes of postoperative morbidity and prolonged hospitalization following surgery, hindering the patient’s early recovery. The frequent concomitant difficulty with oral nutrition further contributes to the patient’s frailty, already compromised by the surgical procedure, increasing the risk of secondary deterioration. Adherence to ERAS protocols is crucial, as it increases the likelihood of early postoperative recovery [14–16]. Both early feeding and early active mobilization, if not implemented, have a negative impact on the restoration of intestinal function. In a previous study a compliance ≥ 85% of ERAS items was associated with a significant reduction in PPOI after colectomies from 46.5 to 26.75% [17]. Moreover, a recent meta-analysis showed that PPOI following colorectal surgery is also associated with increased hospital costs, likely related to prolonged hospital stay and postoperative complications [14, 15].

The recorded incidence of PPOI in the current study was 21.5%, showing that, despite efforts to adopt minimally invasive surgical techniques and advances in perioperative care, PPOI remains a frequent postoperative complication that should not be underestimated and whose prevention and management still require improvement. Goal-directed fluid therapy is an important component of ERAS pathways and may be relevant to postoperative bowel recovery; however, in our exploratory analyses, intraoperative fluid administration did not remain meaningfully associated with PPOI [18–21]. Although these local exploratory findings may provide clinically relevant signals within our cohort, they should not be interpreted as equivalent in evidentiary weight to the external validation analysis, which remains the primary focus of this study. Early postoperative oral feeding and early mobilization are also recommended ERAS components and are considered important for restoration of gastrointestinal function [17–22]. However, delayed oral intake and delayed mobilization may also represent early manifestations or consequences of PPOI rather than true antecedent factors, since patients with evolving ileus often experience nausea, abdominal distension, discomfort, and reduced functional recovery. For this reason, these variables were included only in the fully adjusted exploratory model, and an additional model excluding both temporally ambiguous postoperative variables was performed. Therefore, the associations involving oral intake initiation and autonomous mobilization should be interpreted cautiously and considered hypothesis-generating.

Postoperative opioid use, which is generally minimized within ERAS pathways, was observed in 31.4% of the study population. In the exploratory analyses, opioid use remained associated with PPOI; however, this association should be interpreted cautiously, as opioid administration may also reflect more painful procedures, more difficult postoperative recovery, or greater overall clinical complexity rather than a directly causal effect. Although this proportion was not particularly high, it may still represent an area for further evaluation in perioperative practice. Interestingly, splenic flexure mobilization was associated with lower odds of PPOI in the exploratory model excluding postoperative oral intake initiation and autonomous mobilization. This finding is counterintuitive and not easily explained on clinical grounds, as one might expect greater operative complexity or bowel manipulation to be associated with a higher rather than lower risk of ileus. Given the exploratory nature of the analysis, the limited sample size, and the possibility of residual confounding or case-mix differences, this apparent protective association should be interpreted cautiously and regarded as hypothesis-generating rather than confirmatory.

Finally, patients with PPOI had longer hospital stays (10.5 vs 5.8 days) and longer intensive care unit stays. This association likely reflects the greater overall postoperative burden among patients who develop PPOI, although the direction of this relationship cannot be determined in the present retrospective analysis.

By analyzing specifically the prediction models, in general the ideal algorithm should be well-validated, widely generalizable and easy to use in clinical practice. The two models we evaluated in this study demonstrated poor performance. In our external validation cohort, both models showed poor discrimination (AUC < 0.60) and substantial miscalibration. Calibration slopes below 1 (0.57 for Hain and 0.41 for Wolthuis) indicate attenuation of predictor effects and poor agreement between predicted and observed risks in our population. In addition, Brier scores around 0.16–0.17 reflect limited overall predictive accuracy. These findings suggest that the models do not transport well to our clinical setting, likely due not only to differences in case mix and perioperative management, including ERAS adherence, but also to important differences in outcome definition between the development studies and our validation cohort. Therefore, the present study reflects transportability across both population characteristics and PPOI definition, rather than a pure external validation under identical outcome conditions.

It is essential that predictive models undergo external validation to ensure their reliability and applicability across diverse populations and clinical settings. While internal validation provides important information about the initial robustness of a model, it does not guarantee that the model will perform equally well in different contexts [23]. Therefore, efforts should focus not only on developing new models but also on rigorously validating existing ones externally. As highlighted in a recent systematic review [5], although multiple models for predicting PPOI have been proposed, very few have undergone proper external validation. Strengthening this step is critical to advancing from theoretical tools to clinically useful instruments that can effectively guide perioperative management and improve patient outcomes.

## Limitations and strengths

The current study has several limitations. First, it was conducted at a single center using a retrospective design, which may limit the generalizability of the findings. This external validation therefore represents a single-centre transportability assessment. Given the limited number of events (*n* = 43), performance estimates—particularly discrimination and calibration metrics—were imprecise, as reflected by wide confidence intervals. Additionally, the small sample size and low adherence to certain ERAS items, such as postoperative opioid use, further restrict the robustness of the results. Some clinically relevant variables were not systematically collected in our dataset and therefore could not be assessed. One strength of this study was the use of a standardized definition of PPOI in our cohort, based on Vather’s criteria. In contrast, the two previously which limits comparability. Althoughthisstudywasdesigned as an external validation, the definition of prolonged postoperative ileus differed from that used in the original model development studies. This discrepancy likely contributed to the observed reduction in model performance. Differences in outcome definition are known to substantially influence discrimination and calibration metrics, and therefore our findings should be interpreted with caution.

## Conclusion

PPOI remains a clinically relevant and incompletely resolved postoperative complication. External validation of two existing predictive models demonstrated poor performance in this cohort, highlighting the need for harmonized outcome definitions and broader multicentre validations to identify patients at risk. Accurately identifying high-risk patients who may benefit from targeted preventive strategies remains an important goal and warrants further investigation.

## Data Availability

The data that support the findings of this study are available on request from the corresponding author.
